# Tailored transethosomal systems for tadalafil transdermal delivery: Impact of Phosal and edge activators on skin permeation and cellular uptake

**DOI:** 10.1016/j.ijpx.2025.100376

**Published:** 2025-08-16

**Authors:** Turky Omar Asar, Hossam S. El-Sawy, Ahmed M. Reda, Mohamed Ashraf, Amer H. Asseri, Abdelsattar M. Omar, Tarek A. Ahmed, Khalid M. El-Say

**Affiliations:** a Department of Biology, College of Science and Arts at Alkamil, University of Jeddah, Jeddah, Saudi Arabia; b Department of Pharmaceutics and Pharmaceutical Technology, Faculty of Pharmacy, Egyptian Russian University, Cairo 11829, Egypt; c Department of Pharmaceutics, College of Pharmacy, University of Kut, Wasit 52001, Iraq; d Department of Biochemistry, Faculty of Pharmacy, Egyptian Russian University, Cairo 11829, Egypt; e Department of Laboratory Sciences, College of Pharmacy, University of Kut, Wasit 52001, Iraq; f Department of Biochemistry, Faculty of Science, King Abdulaziz University, Jeddah 21589, Saudi Arabia; g Department of Pharmaceutical Chemistry, Faculty of Pharmacy, King Abdulaziz University, Jeddah 21589, Saudi Arabia; h Department of Pharmaceutics, Faculty of Pharmacy, King Abdulaziz University, Jeddah 21589, Saudi Arabia

**Keywords:** Tadalafil, Phosal-based transethosomes, Box-Behnken design, *Ex vivo* permeation profile, *In vitro* cytotoxicity study, Cellular uptake

## Abstract

Tadalafil (TDLF), a Biopharmaceutics Classification System (BCS) Class II drug, exhibits poor aqueous solubility and extensive first-pass metabolism, which limits its therapeutic efficacy. We developed Phosal-based transethosomes (TrEthOs) to overcome these challenges, thereby enhancing transdermal delivery. A Box-Behnken design was employed to optimize the formulation by evaluating the effects of Phosal type, polyethylene glycol (PEG) 400 concentration, and cholesterol content. The optimized TrEthOs exhibited a mean vesicle size of 129.74 nm and an entrapment efficiency of 67.3%, ensuring efficient encapsulation of the drug. *Ex vivo* permeation studies demonstrated a cumulative TDLF permeation of 70.24% over 6 hours, with a steady-state flux of 19.49 × 10^-4^ mg/cm^2^·min. Confocal laser scanning microscopy confirmed deep skin penetration, while *in vitro* studies revealed significantly enhanced cellular uptake (P < 0.0001) and reduced cytotoxicity (IC₅₀ = 67.61 μg/mL) compared to pure TDLF (IC₅₀ = 34.85 μg/mL). The novel Phosal-based TrEthOs system presents a promising transdermal drug delivery strategy, potentially reducing dosing frequency and improving patient compliance. These outcomes emphasize the potential of advanced nanocarrier systems to optimize systemic bioavailability while minimizing adverse effects.

## Highlights

• Phosal-based transethosomes (TrEthOs) enhanced tadalafil’s transdermal permeability.
• Box-Behnken Design ensured systematic optimization of key permeability attributes.
• CLSM imaging confirmed 3.3-folds superior skin permeability of TDLF-loaded TrEthOs.
• TrEthOs showed 1.94- and 5.64-folds more safety and cellular uptake than pure TDLF.
• Formulation strategy supports controlled release and improved patient adherence.

## Graphical abstract

**Figure:**
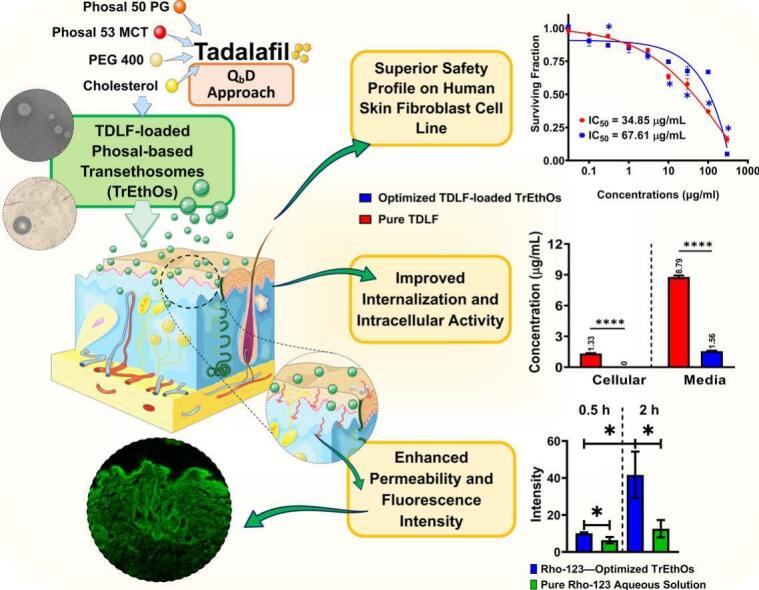

## 1 Introduction

The oral administration of drugs, while being the most conventional route, is often hindered by challenges such as low bioavailability and extensive first-pass metabolism. Such challenges would potentially impact the frequency of dosing, required efficacy and safety, and ultimately, patient compliance. Tadalafil (TDLF), a phosphodiesterase type 5 (PDE5) inhibitor primarily used for treating erectile dysfunction and pulmonary arterial hypertension (Lang et al., 2024), is not immune to these challenges. According to the Biopharmaceutics Classification System (BCS), TDLF is classified as a Class II BCS drug, characterized by low water solubility and high permeability (Alotaibi et al., 2020; Rao et al., 2019; Sohn and Choi, 2024). Although TDLF has gained widespread use due to its longer half-life and reduced side effects compared to other PDE5 inhibitors, such as sildenafil, its pharmacokinetics pose significant obstacles. A major drawback of orally administered TDLF is its relatively poor solubility (from 0.35 to 1.7 μg/mL at 25°C, with log P value of 1.7 ± 0.2) (Baek and Cho, 2016; Choi and Park, 2017; Din et al., 2025; Rao et al., 2019; Wlodarski et al., 2014), leading to delayed absorption and variable plasma concentrations in patients (Alotaibi et al., 2020). This drawback makes designing an alternative delivery system, such as transdermal administration, desirable for overcoming these limitations and enhancing therapeutic efficacy.

Transdermal drug delivery is a promising approach that offers numerous benefits over traditional oral administration. By bypassing the gastrointestinal tract and first-pass hepatic metabolism, transdermal systems can potentially improve the bioavailability of drugs like TDLF, reduce dosing frequency, and improve patient adherence (Prausnitz and Langer, 2008). However, the stratum corneum, the outermost layer of the skin, acts as a significant barrier to the permeation of most drugs, particularly those with large molecular sizes or poor lipid solubility (Ghaferi et al., 2024). Consequently, there has been increasing interest in advanced formulation strategies that enhance the permeability of drugs through the skin, such as ethosomes (EthOs) and transethosomes (TrEthOs) (Ashraf et al., 2023).

EthOs, as described by Touitou et al. (Touitou et al., 2000), are lipid-based vesicular carriers that have gained prominence for enhancing the permeation of hydrophilic and lipophilic drugs through the skin. These vesicles are composed primarily of phospholipids, ethanol, and water, with ethanol playing a crucial role in destabilizing the stratum corneum's lipid bilayer, thereby facilitating the entry of drugs. Besides, EthOs are especially well-suited for the transdermal delivery of drugs like TDLF, which face bioavailability issues when administered orally. Therefore, by encapsulating TDLF within EthOs, the drug can be transported more efficiently through the skin barrier, potentially leading to more consistent plasma levels and a faster onset of action (Mbah et al., 2014). Regarding TrEthOs, they represent a more recent advancement in vesicular drug delivery systems, designed to enhance the benefits of EthOs further. These vesicles incorporate additional edge activators/permeation enhancers, which confer greater elasticity and deformability to the vesicles, allowing them to squeeze through the tight junctions of the stratum corneum more effectively (Raj et al., 2023). This deformability enhances the drug's permeation, providing greater stability and efficiency in delivering both hydrophilic and lipophilic drugs across the skin. Although both TrEthOs and EthOs contain ethanol and enhance skin transport, EthOs may have problems with physical stability. Moreover, TrEthOs possess greater flexibility and deeper permeation relative to liposomes, which are more rigid and have restricted skin penetration. Niosomes composed of non-ionic surfactants are economically advantageous but have lower deformability and entrapment efficiency. Offering a balance of stability, flexibility, and enhanced penetration through the skin, TrEthOs are superior to EthOs, as well as liposomes and niosomes, due to their low skin permeability (Chowdary et al., 2023; Seenivasan et al., 2025). Studies have shown that TrEthOs have superior skin penetration capabilities compared to conventional EthOs and other lipid-based systems, making them an ideal candidate for the transdermal delivery of TDLF (Buya et al., 2024).

To further enhance the solubility and permeability of TDLF, the incorporation of excipients such as Phosal has gained attention. Phosal is a proprietary blend of phospholipids, available in various formulations designed to enhance the solubility and permeability of poorly water-soluble drugs (Ajdary et al., 2020; Duong et al., 2020; Kikwai et al., 2002; Santos Porto et al., 2024; Truszkowska et al., 2024). Phosal 50 PG and Phosal 53 MCT are two such compositions that have shown particular promise in improving transdermal drug delivery. Phosal 50 PG contains phospholipids (∼50%) in combination with propylene glycol, a known penetration enhancer (Hoogevest, 2020). At the same time, Phosal 53 MCT (∼53% phosphatidylcholine) incorporates medium-chain triglycerides (MCT), which serve as an emollient and further promote drug solubility and skin hydration (Duong et al., 2019; Hoogevest, 2020, 2017). The phospholipid composition of Phosal formulations helps to fluidize the lipid bilayers of the stratum corneum, thus facilitating the permeation of drugs like TDLF through the skin (Borges et al., 2024; Hsieh et al., 2022). Additionally, the presence of MCT in Phosal 53 MCT has improved the stability of vesicular carriers and enhanced drug encapsulation (Komeil et al., 2021), making it an ideal excipient for use in TrEthO-based delivery systems. In addition, Polyethylene glycol 400 (PEG 400) was used as an edge activator. PEG 400 increases vesicle flexibility when incorporated into bilayers by disrupting membrane stabilization, which allows vesicles to deform under mechanical stress. Molecular deformability provides obstacle bypassing through narrow pathways between the cells of the stratum corneum. TrEthOs, which comprised PEG 400 as an edge activator, were found to have better skin biocompatibility and superior transdermal delivery of drugs compared to oral administration (Adin et al., 2023; Seenivasan et al., 2025).

TDLF was selected as a model drug due to its low aqueous solubility and lipophilic nature, which present formulation challenges that nanocarrier-based delivery systems can address. However, it exhibits high gastrointestinal permeability (Sohn and Choi, 2024). Its limited solubility restricts its broader application. The transethosomal system was designed to enhance solubilization and improve delivery potential for such poorly water-soluble compounds. To emphasize, the limited TDLF oral bioavailability, resulting from poor water solubility and extensive first-pass metabolism, would lead to variable systemic exposure and inconsistent therapeutic outcomes, necessitating alternative routes of administration. The current study focuses on the transdermal delivery of TDLF as a practical approach to enhance systemic bioavailability and circumvent hepatic metabolism. A transdermal model was employed to investigate drug permeation through excised rat skin, serving as a surrogate for human skin. The goal was to develop a transethosomal system that enhances skin permeability and drug deposition in systemic circulation. This route offers a non-invasive alternative, particularly for patients requiring long-term PDE5 inhibitor therapy in either ED or PAH, aiming to reduce dosing frequency and improve adherence.

Therefore, the combination of Phosal-based TrEthOs can potentially represent a novel and promising approach to overcoming the barriers to transdermal delivery of TDLF. Phosal's ability to improve drug solubility and skin permeation, when coupled with the deformability and penetration-enhancing properties of TrEthOs, is expected to result in an efficient delivery system that enhances the permeability and internalization of TDLF. To date, few studies have explored the incorporation of Phosal into ethosomal or transethosomal systems, making this a novel area of investigation with significant potential to improve the clinical outcomes of TDLF therapy. This study hypothesizes that incorporating Phosal 50 PG and Phosal 53 MCT into TrEthO formulations will significantly enhance the transdermal delivery of TDLF, leading to improved skin permeability and, consequently, enhanced therapeutic outcomes and compliance. The findings of this research could have broad implications for the development of transdermal delivery systems for other poorly soluble and poorly permeable drugs, offering new avenues for improving patient outcomes and adherence in clinical practice. Specifically, the study prepared and characterized different Phosal-based TrEthOs encapsulating TDLF. In addition, a Quality by Design approach evaluated the effect of varying the levels of different types of Phosal and other components of TrEthOs on the permeability attributes. Moreover, the permeation properties of the excised rat skin were further investigated for the optimized TrEthO formulation through confocal and cellular uptake *in vitro* studies.

## 2 Materials and methods

### 2.1 Materials

Polyethylene glycol 400 (PEG 400) was bought from Spectrum Chemical Manufacturing Corporation (Gardena, CA, USA), whilst TDLF was acquired from Jinlan Pharm-Drugs Technology Co., Ltd. (Hangzhou, China). Phosal® 50 PG and Phosal® 53 MCT were procured from Lipoid GmbH (Ludwigshafen, Germany), while Cholesterol and 3-(4, 5-dimethylthiazol -2-yl)-2, 5-diphenyltetrazolium bromide (MTT) were provided from Sigma Aldrich (St. Louis, MO, USA). Potassium phosphotungstate was generously donated as a present to the Pharmaceutical Technology Department of the National Research Centre in Cairo, Egypt. Wako Pure Chemical Industries, Ltd. (Osaka, Japan) was the source of rhodamine 123 (MW 380.8). Merck (Darmstadt, Germany) provided the methanol, ethanol, and acetonitrile. The additional solvents used in this investigation were from Honeywell Riedel-de Haën AG (Seelze, Germany).

### 2.2 Optimization of TDLF-loaded Phosal-based TrEthOs

#### 2.2.1 Design of experiments

In 15 runs, the effects of three factors were examined employing Box-Behnken Design (BBD) in a randomized sequence. The program Statgraphics® Centurion XVI, Version 16.1.11 (Stat-Point, Inc., USA), was used to build a three-factor, 15-run BBD. A preliminary study was conducted before the experimental design to define and identify the upper, mid, and lower values for each element. Table 1 lists the factors discussed in this article, along with their corresponding levels. The response of each dependent variable and optimized conditions could be estimated herein (Ashraf et al., 2024; El-Say et al., 2023). Regarding the use of a non-continuous variable like Phosal type in the Box-Behnken Design (while BBD is traditionally used with continuous numerical variables), literature supports its extension to discrete, ordinal variables when the categories exhibit a clear physicochemical gradient that justifies numerical coding (El-Say et al., 2023; Kassem et al., 2017). In our study, the Phosal type was treated as an ordinal factor, encoded based on relative hydrophilicity, which directly influences vesicle characteristics such as size, drug entrapment, and permeation behaviour. The encoding of levels was thus selected to be –1 for Phosal® 53 MCT (with least hydrophilic physiognomies; rich in medium-chain triglycerides), 0 for Phosal® blend (50 PG:53 MCT, 1:1, with intermediate hydrophilicity), and +1 for Phosal® 50 PG (with highest hydrophilic physiognomies; contains higher content of propylene glycol). This ranking reflects an increasing hydrophilicity gradient based on the ratio of lipophilic (MCT) to hydrophilic (PG) components. The assigned permeability attributes as dependent variables were the mean vesicle size (Y_1_), the entrapment efficiency (Y_2_), the initial amount of TDLF permeated after 0.5 hours (Y3), and the cumulative amount of TDLF permeated after 6 hours. (Y_4_), steady-state flux (Y_5_), permeability coefficient (Y_6_), and diffusion coefficient (Y_7_). Table 2 lists the recorded responses and run composition produced by BBD.

**Table 1:** Independent and dependent variables of Box-Behnken design for TDLF–loaded TrEthOs formulations development.

Independent variables (Factors)	Levels			Units
	Low (-1)	Medium (0)	High (+1)	
X_1_: Phosal type	53-MCT	50-PG: 53-MCT (1:1)	50-PG	–
X_2_: PEG 400 concentration	0	15	30	%
X_3_: Cholesterol concentration	0	5	10	%
Dependent variables (Responses)	Units		Goal	
Y_1_: Mean vesicle size	nm		Minimize	
Y_2_: Entrapment efficiency	%		Maximize	
Y_3_: Initial permeation (0.5 h)	%		Maximize	
Y_4_: Cumulative permeation (6 h)	%		Maximize	
Y_5_: Steady State Flux (*J*_*ss*_)	(mg/cm^2^.min) × 10^-4^		Maximize	
Y_6_: Permeability Coefficient (*Pc*)	(cm/min) × 10^-4^		Maximize	
Y_7_: Diffusion Coefficient (*D*)	(cm^2^/min) × 10^-4^		Maximize

**Table 2:** Experimental runs and observed values of responses for BBD.

Runs	Factors^a^			Responses^b^						
	X_1_	X_2_	X_3_	Y_1_	Y_2_	Y_3_	Y_4_	Y_5_	Y_6_	Y_7_
		*(%)*	*(%)*	*(nm)*	*(%)*	*(%)*	*(%)*	*(mg/cm*^*2*^*.min) × 10*^*-4*^	*(cm/min) × 10*^*-4*^	*(cm*^*2*^*/min) × 10*^*-4*^
F1	50-PG: 53-MCT (1:1)	30	0	395.25	66.15	29.96	70.36	14.16	7.0786	5.6
F2	50-PG	30	5	123.33	57.66	34.4	76.2	17.782	8.891	9.367
F3	53-MCT	0	5	711.92	76.27	22.08	64.3	16.125	8.0624	7.608
F4	50-PG	15	10	142.42	53.68	35.21	77.23	17.466	8.7332	8.633
F5	50-PG: 53-MCT (1:1)	30	10	264.67	91.85	18.24	60.67	16.046	8.023	7.462
F6	50-PG: 53-MCT (1:1)	0	10	535.58	67.86	29.35	71.17	14.157	7.079	5.6
F7	50-PG	15	0	313.08	47.81	44.7	87.94	19.576	9.788	9.108
F8	53-MCT	15	0	709.92	75.77	21.88	63.7	15.12	7.56	6.4426
F9	53-MCT	15	10	476	92.84	14.4	56.83	15.846	7.923	6.781
F10	53-MCT	30	5	400.25	94.6	16.22	58.85	16.251	8.1255	6.9207
F11	50-PG: 53-MCT (1:1)	0	0	541.5	56.21	42.49	84.3	18.187	9.0935	8.2904
F12	50-PG	0	5	289.83	52.24	45.92	90.57	19.95	9.9748	9.0262
F13	50-PG: 53-MCT (1:1)	15	5	511.08	65.83	29.76	71.58	12.937	6.4683	4.3035
F14	50-PG: 53-MCT (1:1)	15	5	498.58	65.7	30.57	71.37	12.279	6.1395	4.1219
F15	50-PG: 53-MCT (1:1)	15	5	555.58	64.93	29.35	71.78	11.79	5.8949	3.9805

Abbreviations:

X_1_ is the Phosal type; X_2_ is the PEG 400 concentration (%) and X_3_ is Cholesterol concentration (%). Y_1_ is the mean vesicle size (nm); Y_2_ is the entrapment efficiency percentage (%); Y_3_ is the percentage of TDLF initial permeation after 0.5 h (%); Y_4_ is the percentage of TDLF cumulative permeation after 6 h (%); Y_5_ is the Steady State Flux (*J*_*ss*_; mg/cm^2^.min × 10^-4^); Y_6_ is the Permeability Coefficient (*P*_*c*_; cm/min × 10^-4^); and Y_7_ is the Diffusion Coefficient (*D*; cm^2^/min × 10^-4^).

a Total weight of Phosal included in each TrEthOs formulation (regardless of the type used) = 100 mg. PEG 400 and cholesterol percentages correspond to the weight of the incorporated Phosal (100 mg) to each TrEthOs formulation.

b Values of responses are the means of triplicate measurements for each response, and SD values did not exceed 5% of the stated values.

#### 2.2.2 Preparation of different TDLF-loaded Phosal-based TrEthOs

TDLF-loaded TrEthOs formulations were created using the thin-film hydration technique with modifications (El-Sonbaty et al., 2022). In a round-bottom flask, 20 mg of TDLF, along with the specified amounts of Phosal® 50 PG, Phosal® 53 MCT, PEG 400, and cholesterol (according to their amounts in each formulation as listed in Table 2), were dissolved in 10 mL of chloroform. The total Phosal weight included in each TrEthOs formulation (regardless of the type) was 100 mg. PEG 400 and cholesterol percentages corresponded to the weight of the incorporated Phosal (100 mg) in each TrEthOs formulation. Using a Büchi-M/HB-140 rotary evaporator (Flawil, St. Gallen, Switzerland) installed with a RE3022C vacuum pump (Fisher Scientific, Leicestershire, UK), chloroform was then removed at 60-65 °C under vacuum till the formation of a thin layer of lipid film containing TDLF on the interior surface of the round-bottom flask. Afterward, a 1-hour rehydration of the formed lipid film with the hydroethanolic solution (30% v/v) was performed using a magnetic stirrer. The round-bottom flask was additionally connected with a glass condenser cooled at 7 °C using a cooling thermostatic water bath. The rehydration stage was carried out for 1 h at room temperature to ensure no ethanol evaporated. To ensure proper swelling, the resulting dispersion was refrigerated. A probe sonicator (UP100H, Hielscher Ultrasonics GmbH, Berlin) was used to sonicate the produced dispersions at 4 °C for 4 min to produce nanosized vesicles. Employing an ice jacket to maintain a sonication temperature of 4 °C, the sonication cycle was completed at 70% amplitude for 4 min, with a pulse of 10 s off and 50 s on. All prepared dispersions were then placed in the refrigerator until further investigations.

### 2.3 Characterization of the prepared TDLF-loaded TrEthOs

#### 2.3.1 Zeta potential, vesicular size, and polydispersity index

The vesicular size and zeta potential of the TDLF-loaded TrEthOs formulations were measured using dynamic light scattering (DLS). By tracking the intensity of light scattered from the particles to a detector over time, the DLS method can be used to estimate the size of particles and vesicles in a liquid. Brownian motion causes the particles to move, and when two or more of them scatter light, it can interfere with the detector either positively or negatively. Vesicular sizes ranging from 0.3 nm to 10 μm can be determined by calculating the autocorrelation function of light intensity and assuming particle dispersion. The zeta potential of nanodispersions can also be detected using this device (Elimam et al., 2024). The homogeneity of the produced vesicles was also examined by measuring the polydispersity index (PDI). DLS equipment (DLS Zetasizer Nano ZPS Instrument; Malvern, Worcestershire, UK) was used to perform the measurements at 25 °C using laser diffraction.

#### 2.3.2 Entrapment efficiency

To produce a nanovesicular dispersion devoid of any unentrapped TDLF, a predetermined amount of lipidic mixtures was centrifuged in a cooling centrifuge (Centurion Scientific Ltd., Stoughton, UK) for 1 h at -2 °C and 10,000 rpm.

After washing the centrifuged suspension to remove any free TDLF, a second centrifugation cycle was performed to obtain a lipidic suspension pellet (El-Say et al., 2024). After the separation and washing procedures, the pellets formed from the previously washed vesicles were redispersed in 10 mL of distilled water. One milliliter of the re-dispersed vesicles was then dissolved in 30 ml of methyl alcohol and sonicated for around 10 min. The quantity of entrapped TDLF in nanovesicles was then measured at a wavelength of 298 nm using a spectrophotometric technique with a UV-visible spectrophotometer (Jasco V-630, Tokyo, Japan) (Gardouh et al., 2021; Megahed et al., 2022). TDLF-free nanovesicles were used as a blank and were prepared using the same procedure as that for TDLF-loaded TrEthOs. To minimize error, each batch was prepared in triplicate, and the mean absorbance of TDLF-free nanovesicles was subtracted to ensure accuracy. Finally, the following equation (Eq. 1) was used to determine the entrapment effectiveness percentage of TDLF.

(1) $$\text{Entrapment efficiency}\;\left(\%\right)=\frac{\text{Amount of TDLF entrapped}\;}{\text{Total amount of TDLF}}\times 100$$

#### 2.3.3 Ex vivo *permeation study*

The effectiveness of the proposed formulations in enhancing the transdermal permeation of TDLF-loaded TrEthOs *via* rat skin was examined in an *ex vivo* permeation investigation adopted from a previous study with modifications (Abdellatif et al., 2017). Rat skin was prepared for *ex vivo* research in accordance with the following ethical approval by the Egyptian Russian University Faculty of Pharmacy's Ethical Committee (ERUFP-PT-24-001). The handling of animals and the *ex vivo* studies have been conducted in compliance with Animal Research: Reporting of In Vivo Experiments (ARRIVE) guidelines.

Additionally, the study was conducted in accordance with the National Institutes of Health Guide for the Care and Use of Laboratory Animals (NIH Publication No. 8023, revised 1978). In short, the Nile for Pharmaceuticals and Chemical Industries, Cairo, Egypt, supplied male Wistar rats, 4–5 weeks old, weighing between 80 and 120 g. The animals had unrestricted access to water. General conditions and the environment were closely monitored. Following euthanasia, an electric clipper was used to remove the skin hair. Afterward, a surgical blade was used to separate the dorsal side of the skin, and a scalpel was used to carefully remove the subcutaneous fat and connective tissues. After being cleaned with 0.9% sodium chloride, the skin's integrity was assessed. Before being used for investigation within four weeks, the trimmed skin was divided into suitable round pieces and wrapped in aluminum foil, then stored in an Ultra-Low-Temperature Freezer at -80 °C (Daihan, WUF-25, Daihan Scientific Co., Ltd., Korea).

A locally constructed Franz's diffusion cell with a 10 mL receiver compartment and an effective area for permeation of 1.30 cm^2^ was used to investigate the *ex vivo* skin penetration of TDLF-loaded TrEthOs. To stop formulations from evaporating, a screw cover was included for the donor chamber. The prepared skin piece was secured between the two compartments, with the dermal side of the skin facing the diffusion cell's receiver compartment. As receiving media, sodium dodecyl sulfate (0.05% w/v) in PBS (pH 5.8 ± 0.2) was used, adhering to the sink condition (Raposo et al., 2015). The media was continuously stirred at 700 rpm and thermostated at 37 ± 0.5 °C. At 0, 0.5, 1, 1.5, 2, 3, 4, and 6 h intervals, samples of 200 μL volume were taken from the receiver compartment, while 1 mL of TrEthOs formulation, equivalent to 2 mg of TDLF, was added to the donor compartment. Each collected sample was replaced with an equal volume of pre-warmed medium (Gardouh et al., 2021; Megahed et al., 2022), which was then examined for the amount of penetrated TDLF at a wavelength of 298 nm using the aforementioned spectrophotometric approach with a UV-visible spectrophotometer (Jasco V-630, Tokyo, Japan). TDLF-free (blank) TrEthOs were prepared and underwent the same procedure and conditions as for TDLF-loaded TrEthOs in the study. The permeated TDLF amounts (mg/cm^2^) against time profiles were plotted to determine the *ex vivo* permeation parameters. The steady-state flux (*J*_*ss*_) was calculated from the slope of the produced plots, and the permeability coefficient (*Pc*), which is the ratio of *J*_*ss*_ to the initial drug load (*C*_*0*_), was measured. As indicated by the following equation (Eq. 2), the diffusion coefficient (*D*) was calculated by plotting the total amount of TDLF absorbed through constant/unit area as a function of the square root of time (√t) (El-Say et al., 2017, El-Say et al., 2024).

(2) $$D={\left(\frac{\mathrm{Slope}}{2C_{0}}\right)}^{2}\times \pi \;\text{Higuchi model}$$

This equation is derived from Fick’s second law of diffusion and is commonly used in *ex vivo* permeation studies involving biological membranes. Although its mathematical structure resembles that of the Higuchi model used in *in vitro* release studies, its application and interpretation are different. In our study, the goal was to estimate drug permeation behaviour across *ex vivo* rat skin, not to analyse drug release from a formulation into a synthetic medium. Therefore, release kinetics models such as Higuchi were not applied, as they are not suitable for the experimental system used. Thus, R^2^ values related to release model fitting were not calculated or reported in related Figures, since the data were not intended for kinetic model evaluation.

### 2.4 Prediction and assessment of the optimized TDLF-loaded TrEthOs

The findings required to choose the optimized formulation were obtained by measuring the various response variables for the fifteen developed TrEthOs formulations. To forecast the composition of the optimized formulation for all response variables combined, the software conducted a desirability study and multiple-response optimization. The optimized formulation was then selected based on the desirability index obtained from the software analysis. To compare the expected variable responses with the optimized formulation, the seven response variables were measured using the same procedures as those for the fifteen formulations, which were formulated in accordance with BBD. Further characterizations for the optimized TDLF-loaded TrEthOs have been performed, including morphological examination, Fourier transform infrared spectroscopy (FTIR), and differential scanning calorimetry (DSC).

#### 2.4.1 Optical and transmission electron microscope examination

Optical microscopy (bright-field) was used to analyze the morphological features of the optimized TDLF-loaded TrEthOs formulation. The nanovesicular formulations were examined using an optical microscope (Leica DM300, Wetzlar, Germany), equipped with a 16× eyepiece and a 40× lens. A digital camera (iPhone 12, Apple, USA) was used to take photomicrographs (Desoqi et al., 2021). The produced nanovesicular formulations were examined morphologically using a transmission electron microscope (TEM). A drop of the nanovesicular suspension was applied to a carbon-coated grid, followed by the addition of a 1% potassium phosphotungstate negative staining solution and drying. The JEOL (JEM-100CX; Japan) TEM was then used to take micrographs at an accelerating voltage of 200 kV (Al-Zuhairy et al., 2023).

#### 2.4.2 Physicochemical characterization using FTIR and DSC analysis

FTIR spectroscopy was performed using an FTIR Spectrometer (IR Affinity-1S FTIR, Japan) to assess potential chemical interactions between TDLF and the excipients of the optimized TrEthOs formulation, by detecting characteristic functional group vibrations and shifts in peak positions. A suitable quantity of potassium bromide was added to each sample of the optimized formulation and pure TDLF before they were compressed into discs. Every disc was then positioned in a light path and scanned over a wavenumber range of 4000–500 cm^−1^ at a speed of 4 mm/s and a resolution of 2 cm. The interaction between the components of the optimized formulation and the pure TDLF was further examined using DSC. The Shimadzu DSC-60 Differential Scanning Calorimeter (Shimadzu Corporation, Kyoto, Japan) was used to obtain DSC thermograms. Samples were heated at a rate of 5 °C/min over a temperature range of 25 to 400 °C, with a nitrogen purge flow rate of 50 mL/min and a protective gas flow rate of 20 mL/min. Methods used for FTIR and DSC analysis were in accordance with previous studies (El-Say et al., 2023; Gardouh et al., 2021).

### 2.5 Confocal laser scanning microscope

Using the fluorescent dye rhodamine-123 (Rho-123), a confocal laser scanning microscopy (CLSM) analysis was performed to investigate the optimized transdermal permeation of TrEthOs. While preparing the optimized TrEthOs, a precise quantity of Rho-123 equal to 0.0005% w/v was introduced in place of TDLF. As mentioned in "section 2.4.3," 1 mL of the optimized Rho-123-loaded TrEthOs was added to Franz's diffusion cell. After 0.5 h, the skin was cut with a cryostat microtome to verify fluorescence penetration and rinsed with distilled water after removing the residual sample. The skin was fixed in liquid nitrogen until it was examined within 12 hours after being placed on a microscopic slide. The same procedures were repeated for the skin removal after 2 h. Similarly, Rho-123 aqueous solution was added as a control. The skin segments were exposed to the Rho-123 and the optimized Rho-123-loaded TrEthOs for the two time durations specified above. After that, imaging was performed using the CLSM (Leica, TCS SP8, Heidelberg, Germany), which has an excitation wavelength of 573 nm and an emission wavelength of 591 nm. The intensity of the Rho-123 fluorescence in the produced photos was examined using LAS X Core 3.7.4 software (Leica, TCS SP8, Heidelberg, Germany) (AbdelSamie et al., 2016; El-Say et al., 2024; Jouan et al., 2016). For our analysis, each image was acquired under identical gain, exposure, and laser power settings to ensure consistency. Z-stack projection was avoided, and only single optical sections were used to maintain accurate comparisons across samples. The quantification steps included manual selection of a standardized ROI at a defined depth (epidermis and dermis), mean fluorescence intensity (MFI) measurement within ROI for each replicate (n = 3), and afterwards exporting numerical values for statistical analysis.

### 2.6 *In vitro* cytotoxicity and cellular uptake studies of the optimized formulation

An *in vitro* cell viability/cytotoxicity study was conducted to determine the IC_50_ value (the concentration that produces 50% inhibition of cell growth) of the optimized TDLF-loaded TrEthOs compared with the pure TDLF drug. These values have been utilized for the subsequent cellular uptake investigation. These studies aim to provide insights into the optimized formulation's activity, cellular internalization ability, and safety. The solvent used to prepare the stock solution of pure TDLF in both the MTT assay and the cellular uptake study was dimethyl sulfoxide (DMSO).

#### 2.6.1 Cell culture

Nawah Scientific Inc., located in Mokatam, Cairo, Egypt, provided the Human Skin Fibroblast (HSF) cell line. The cells were maintained at 37 °C in a humidified, 5% (v/v) CO2 atmosphere in high-glucose DMEM medium (Invitrogen/Life Technologies) supplemented with 100 μg/mL streptomycin, 100 units/mL penicillin, and 10% heat-inactivated fetal bovine serum (Hyclone).

#### 2.6.2 MTT assay for evaluating the in vitro cytotoxicity

The cytotoxicity study was performed utilizing an MTT assay. After seeding 96-well plates with aliquots of 100 μL cell suspension (5×10^3^ cells), the plates were cultured in complete medium for 24 h at 37 °C with 5% CO_2_. Another aliquot of 100 μL of media with either pure TDLF solution or the optimized TDLF-loaded TrEthOs at different concentrations (0.03, 0.1, 0.3, 1, 3, 10, 30, 100, and 300 μg/mL) was used to treat the cells. Following a 48-hour drug/formulation exposure period, the media was discarded, and 100 μL of PBS was added to each well. The wells were then mixed with 20 μL of 1 mg/mL MTT solution and incubated for 4 hours at 37 °C. After that, 100 μL of pure DMSO was used to dissolve the formed formazan crystals. The absorbance of formazan solutions was measured using a multi-well plate reader (BMGLABTECH FLUOstar Omega, Germany) at λ_max_ 570 nm (Kassem et al., 2017; Megahed et al., 2022). In addition, a plain TrEthOs was included in the study as a positive control to exclude any activity that may be raised from the unloaded TrEthOs. A control group treated with DMSO only was also included in the MTT assay.

#### 2.6.3 Quantitative analysis of cellular uptake using HPLC

The cellular uptake of both the optimized TDLF-loaded TrEthOs formulation and pure TDLF was measured. A T25 flask (Greiner Bio-One, Germany) was used to seed cells and incubated for 24 h. Next, two sets of plates were separated. Set I received a 50 μg/mL concentration of pure TDLF, whereas Set II received an optimized TDLF-loaded TrEthOs treatment (50 μg/mL). After 4 h of incubation with pure TDLF or the optimized TDLF-loaded TrEthOs formulation, the treatment medium was aspirated, and the cells were washed twice with cold PBS (pH 7.4) to eliminate extracellular drug. The washed cells were then trypsinized, collected by centrifugation, and subjected to lysis for the quantification of intracellular tadalafil. It is worth mentioning that cells with an 80–90% confluence were harvested using trypsin/EDTA. The cell pellet and 3 mL of the media were stored at -20 °C prior to HPLC analysis (Gardouh et al., 2021).

To collect the sample from the cell pellets, the cells were gently tapped repeatedly on the Eppendorf tube until they were evenly distributed throughout the fluid. To extract the sample, 150 μL of cell pellet suspension was combined with 500 μL of acetonitrile, 250 μL of acetone, and 100 μL of saturated zinc sulphate solution. The combination was then sonicated for 20 minutes and centrifuged at 14,000 rpm for 10 minutes at 4 °C. After that, 10 μL of the clear supernatant was added to the HPLC system and placed into an autosampler vial. To quantify TDLF in the media, 3 mL of the previously collected medium was filtered through a PTFE filter and then directly injected into the HPLC apparatus.

A Waters 2690 Alliance HPLC system with a Waters 996 photodiode array detector (Waters, Milford, MA, USA) was used for the study analysis. Inertsil C_18_ was used for the separation (5μm, 4.6×250 mm). Acetonitrile and 20 mM potassium dihydrogen phosphate buffer (pH 7) made up the mobile phase (50:50, v/v), which was pumped at a rate of 1 mL/min. The whole run time was 12 minutes. The wavelength at which the quantification was done was 290 nm. To create the calibration curve, a stock solution of 100 μg/mL TDLF dissolved in acetonitrile was used. Acetonitrile was also used to dilute the stock solution, resulting in working solutions with concentrations ranging from 0.2 to 10 μg/mL. Following the examination of working standard samples using the previously indicated HPLC procedure, the concentration of TDLF in cell pellets and medium samples was calculated using the equation obtained from the calibration curve. After internal testing, the chromatographic conditions used in this investigation were validated and revealed good validation parameters with linearity (R^2^ = 0.9992), accuracy (recovery 95–105%), precision (RSD < 2%), and LOD/LOQ (LOD ≈ 0.39 μg/mL; LOQ ≈ 1.19 μg/mL), which were found to be reliable, accurate, sensitive, selective, and precise.

### 2.7 Statistical analysis

The statistical analysis of the collected data was conducted using GraphPad Prism software, version 8.4.2 (San Diego, CA, USA). Two-way ANOVA and Sidak's multiple comparisons test were used to compare each mean at all points to determine the significance between groups in the *in vitro* cytotoxicity study results. Results are deemed significant if the P-value is less than 0.05. A one-way ANOVA/Tukey–Kramer post-hoc test was used to analyze the results of the cellular uptake investigation at a significance level of P < 0.05. The data are shown as mean ± SD.

## 3 Results and discussion

### 3.1 Optimization of TDLF-loaded Phosal-based TrEthOs

The 3-factor, 3-level BBD model was utilized to optimize TDLF-loaded Phosal-based TrEthOs using data from preliminary research. The selected components' quadratic and interaction impacts on the responses were examined, enhanced, and assessed (Table 1). BBD has been selected as the optimization strategy for our study. This is because BBD is more effective at modeling nonlinear responses with fewer experimental runs than full factorial or central composite designs, especially when dealing with three independent variables. Besides, BBD is ideally suited for investigating interaction effects and quadratic response surfaces while preserving experimental economy and safety. This is particularly important in formulation work involving expensive or delicate/sensitive materials.

The Phosal type used in formulating TrEthOs was employed in this design as X_1_ (depending on the degree of hydrophilicity) with levels in terms of Phosal 53 MCT, Phosal 50-PG, and Phosal 53-MCT (1:1) with Phosal 50 PG. The levels of Phosal Type (X₁) were chosen based on their compositional differences (hydrophilicity) and previously reported effects on vesicle properties. The selected levels vary in hydrophilicity and surface activity, which are known to impact vesicle structure, drug entrapment, and transdermal penetration. Additionally, the PEG 400 concentration levels (X2) were 0%, 15%, and 30%. The selected range of PEG 400 Concentrations was based on its known dual role as a cosurfactant and a permeability enhancer. PEG 400 alters membrane fluidity and affects vesicle deformability, which directly influences skin transport. In addition, 0, 5, and 10% were the cholesterol percentage concentration levels, assigned as X_3_, to examine its stabilizing impact on vesicle bilayers. Cholesterol modulates membrane rigidity and leakage. Therefore, optimizing its level is critical to balance vesicle stability versus permeability.

The selected design generated 15 runs, including center points, which allowed robust statistical analysis of quadratic and interaction terms with minimal confounding. Table 2 displays the results of measuring several response factors for each BBD formulation. Additionally, polynomial equations were generated for every response. It is worth noting that the selection of TDLF, a BCS Class II drug, was intended to assess the system’s ability to improve the handling and delivery of low-solubility molecules. While TDLF is highly permeable through intestinal membranes, this property does not guarantee efficient penetration through other biological barriers. Including both phospholipids and edge activators served distinct roles: phospholipids supported vesicle integrity and drug loading, while edge activators improved flexibility and membrane penetration. These components acted synergistically to enhance delivery, rather than as redundant enhancers.

### 3.2 Characterization of the TDLF-loaded Phosal-based TrEthOs

The characterization of TDLF-loaded TrEthOs was conducted *via* measuring zeta potential, PDI, and permeability attributes assigned for the BBD design including mean vesicle size (Y_1_), entrapment efficiency percentage (Y_2_), percentage of initial amount of TDLF permeated after 0.5 h (Y_3_), percentage of cumulative amount of TDLF permeated after 6 h (Y_4_), steady-state flux (Y_5_), permeability coefficient (Y_6_), and diffusion coefficient (Y_7_).

The recorded values of zeta potential for BBD TDLF-loaded TrEthOs ranged from -21.6 to -26.29 mV for F8 and F7, respectively. In contrast, PDI values ranged from 0.319 to 0.667 for F2 and F8, respectively, indicating that all formulated TrEthOs systems exhibit satisfactory homogeneity and zeta-nano stability features (Elimam et al., 2024). The recorded values of both zeta potential and PDI were also in accordance with what was recorded in previous research works that utilized Phosal 50-PG and Phosal 53-MCT in formulating different nanosystems (Allam et al., 2015; Hsieh et al., 2022). The mean vesicle sizes of TrEthOs formulations demonstrated a range from 123.33 to 709.92 nm for F2 (prepared with Phosal 50-PG and 30% PEG 400) and F8, respectively (Table 2). In addition, the entrapment efficiency of formulated TrEthOs ranged from 47.81 to 94.6% for F7 and F10, respectively. Additionally, the initial TDLF permeation rate after 0.5 h ranged from 14.4% for F9 to 45.92% for F12. In comparison, the cumulative amount of TDLF permeated after 6 h ranged from 56.83% for F9 to 90.57% for F12. Thus, F12 TDLF-loaded TrEthOs (prepared with Phosal 50-PG) showed the highest initial and cumulative permeation compared to other formulations, as reflected in the values of steady-state flux, permeability coefficient, and diffusion coefficient, which were found to be at their highest for F12 (Table 2). In conclusion, the prepared BBD TDLF-loaded TrEthOs had acceptable homogeneity and vesicular stability properties. On the other hand, all selected permeability attributes have displayed diversity in ranges between TrEthOs formulations of different Phosal types and amounts of other compositions. The following section provides further insight into the quantitative estimated effects of factors on BBD responses, which represent the selected permeation parameters.

#### 3.2.1 Statistical analysis of factors' effect

ANOVA was utilized to describe the relevance-focused mathematical treatment of the chosen factors and the polynomial equations that represented the observed responses. In addition to the ANOVA-calculated P-values of the estimates for the TDLF-loaded TrEthOs' researched responses, Table 3 displays the estimated influences of the variables for each of the examined responses. If a factor's effect value is not equal to zero and the accompanying P-value is equal to or less than 0.05, it will be considered a significant effect. Furthermore, a factor's antagonistic effect is shown by a negative sign, whereas a positive sign indicates its synergistic effect.

**Table 3:** Estimated effects of factors associated with P-values for responses (Y_1_-Y_7_).

Factors	Y_1_		Y_2_		Y_3_		Y_4_		Y_5_		Y_6_		Y_7_	
	Factor effect	*p*-value	Factor effect	*p*-value	Factor effect	*p*-value	Factor effect	*p*-value	Factor effect	*p*-value	Factor effect	*p*-value	Factor effect	*p*-value
X_1_	−357.35	0.0001⁎	−32.02	0.0000⁎	21.41	0.0000⁎	22.07	0.0000⁎	2.86	0.0002⁎	1.43	0.0002⁎	2.1	0.0000⁎
X_2_	−223.83	0.0012⁎	14.42	0.0010⁎	−10.25	0.0003⁎	−11.06	0.0000⁎	−1.05	0.0141⁎	−0.52	0.0141⁎	−0.29	0.0890
X_3_	−135.27	0.0102⁎	15.08	0.0008⁎	−10.46	0.0003⁎	−10.1	0.0001⁎	−0.88	0.0264⁎	−0.44	0.0264⁎	−0.24	0.1439
X_1_^2^	−164.31	0.0212⁎	1.71	0.6032	−1.2	0.5195	0.71	0.5731	6.56	0.0000⁎	3.28	0.0000⁎	5.1	0.0000⁎
X_1_X_2_	72.583	0.1884	−6.46	0.0815	−2.83	0.1488	−4.44	0.0109⁎	−1.15	0.0351⁎	−0.57	0.0351⁎	0.51	0.0478⁎
X_1_X_3_	31.63	0.5365	−5.61	0.1178	−1.01	0.5691	−1.92	0.1495	−1.42	0.0165⁎	−0.71	0.0165⁎	−0.41	0.0942
X_2_^2^	−116.52	0.0657	7.7	0.0551	0.72	0.6923	1.11	0.3872	3.83	0.0003⁎	1.91	0.0003⁎	3.09	0.0000⁎
X_2_X_3_	−62.33	0.2480	7.02	0.0643	0.71	0.6876	1.72	0.1883	2.96	0.0007⁎	1.48	0.0007⁎	2.28	0.0001⁎
X_3_^2^	−58.48	0.2917	2.37	0.4781	−0.49	0.7886	−1.01	0.4287	2.78	0.0011⁎	1.39	0.0011⁎	2.11	0.0001⁎
R^2^	0.97474		0.98598		0.98996		0.99567		0.99077		0.99078		0.99593	
P-value	9.06E-12		1.96E-13		2.24E-14		9.44E-17		1.29E-14		1.29E-14		6.30E-17	
PRESS	157190.5142		699.2709		209.7981		99.87395		3.7012		0.92344		2.39216	

Abbreviations:

X_1_ is the Phosal type; X_2_ is the PEG 400 concentration (%); and X_3_ is Cholesterol concentration (%). X_1_X_2_; X_1_X_3_; X_2_X_3_ are the interaction terms between the factors. X_1_^2^, X_2_^2^, X_3_^2^ are the quadratic terms of the factors. Y_1_ is the mean vesicle size (nm); Y_2_ is the entrapment efficiency percentage (%),Y_3_ is the percentage of TDLF initial permeation after 0.5 h (%); Y_4_ is the percentage of TDLF cumulative permeation after 6 h (%); Y_5_ is the Steady State Flux (*J*_*ss*_; mg/cm^2^.min × 10^-4^); Y_6_ is the Permeability Coefficient (*P*_*c*_; cm/min × 10^-4^); and Y_7_ is the Diffusion Coefficient (*D*; cm^2^/min × 10^-4^).

⁎ Significant effect of factors on individual responses.

According to the revealed outcomes, the Phosal type used in formulating TrEthOs (X_1_) was found to have a significant antagonistic influence on the mean vesicle size (Y_1_) with P = 0.0001, and the entrapment efficiency percentage (Y_2_) with P < 0.0001. On the other hand, X_1_ was found to exert a significant synergistic effect on the percentage of the initial amount of TDLF permeated after 0.5 h (Y_3_), the percentage of the cumulative amount of TDLF permeated after 6 h (Y_4_), steady-state flux (Y_5_), permeability coefficient (Y_6_), and diffusion coefficient (Y_7_), with P < 0.0001, P < 0.0001, P = 0.0002, P = 0.0002, and P < 0.0001, respectively. Besides, PEG 400 percentage concentrations (X_2_) were found to exhibit a significant synergistic effect on Y_2_ with P = 0.001 while exerting a significant antagonistic impact on Y_1_, Y_3_, Y_4_, Y_5_, and Y_6_, at P-values of 0.0012, 0.0003, < 0.0001, 0.0141, and 0.0141, respectively. In addition, the cholesterol percentage concentration (X_3_) was found to have a significant positive influence on Y_2_ (P-value of 0.0008), while exhibiting a significant negative effect on Y_1_ (P-value of 0.0102), Y_3_ (P-value of 0.0003), Y_4_ (P-value of 0.0001), Y_5_ (P-value of 0.0264), and Y_6_ (P-value of 0.0264). Furthermore, it was observed that the quadratic effect of X_1_ (X_1_^2^) negatively influenced Y_1_ with a P-value of 0.0212 while positively impacting Y_5_, Y_6_, and Y_7_ (P < 0.0001 for the three responses). Moreover, Y_4_, Y_5_, and Y_6_ were influenced antagonistically (0.0109, 0.0351, and 0.0351 P-values, respectively) along with the positively impacted Y_7_ (P = 0.0478) by the interaction term (X_1_X_2_). Additionally, the interaction term (X_1_X_3_) negatively influenced Y_5_ and Y_6,_ with a P-value of 0.0165 for both responses. In addition, it was detected that the quadratic term of X_2_ (X_2_^2^) had a significant synergistic effect on Y_5_, Y_6_, and Y_7_ with (P = 0.0003, P = 0.0003, and P < 0.0001, respectively). Also, a significant synergistic effect on Y_5_, Y_6_, and Y_7_ was demonstrated by the interaction term (X_2_X_3_; P = 0.0007, P = 0.0007, and P = 0.0001, respectively) as well as the quadratic term of X_3_ (X_3_^2^; P = 0.0011, P = 0.0011, and P = 0.0001, respectively).

#### 3.2.2 Influences on mean vesicular size (Y_1_)

**Fig. 1:**
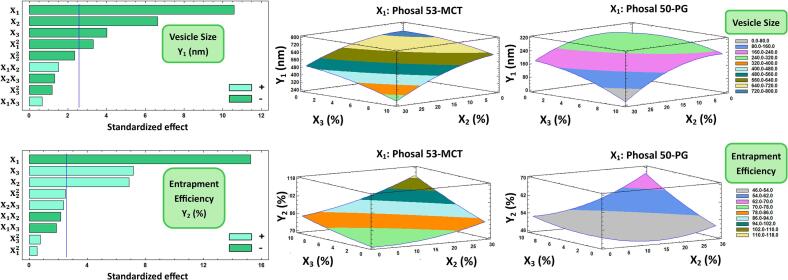
Left panel: Pareto chart displaying the factors' standardized influence on the observed responses (Y_1_ and Y_2_). Mid and right panel: Plots of contour-response surfaces illustrating how variables affect the observed responses (Y_1_ and Y_2_). The TDLF-loaded Phosal 50 PG-based TrEthOs exhibits lower ranges in both vesicle size and entrapment efficiency compared to the TDLF-loaded Phosal 53 MCT-based TrEthOs. Note: X_1_ is the Phosal type, X_2_ is the PEG 400 concentration (%), and X_3_ is Cholesterol concentration (%). X_1_X_2_, X_1_X_3_, X_2_X_3_ are the interaction terms between the factors. X_1_^2^, X_2_^2^, X_3_^2^ are the quadratic terms of the factors.

The mean vesicle sizes (Y_1_) of the BBD TDLF-loaded TrEthOs ranged from 123.33 to 709.92 nm for F2 and F8, respectively (Table 2). It was found that the Phosal type used in formulating TrEthOs (X_1_), PEG 400 percentage concentrations (X_2_), and cholesterol percentage concentration (X_3_) have significant inverse/antagonistic effects on Y_1_. As demonstrated in the Pareto chart of Y_1_ (Fig. 1), X_1_ was noted to have the main antagonistic effect on Y_1_. At fixed levels of X_2_ and X_3_ in F7 and F8, respectively, it was noticed that changing X_1_ from Phosal 50 PG to Phosal 53 MCT (decreasing hydrophilicity) was associated with the increase of Y_1_ from 313.08 to 709.92 nm, respectively (Table 2). On the other hand, the change of X_1_ from Phosal 53 MCT to Phosal 50 PG (increasing hydrophilicity) in F9 and F4, respectively, was evidenced by the decrease of Y_1_ from 476 to 142.42 nm, respectively, at constant levels of X_2_ and X_3_. An inverse/antagonistic relationship was also observed between the quadratic effect of X_1_ (X_1_^2^) and Y_1_.

The presence of 40% propylene glycol (PG) in the composition of Phosal 50 PG can be considered a rational and logical cause of the previous finding. As noted from the contour response surface plot in Fig. 1, TDLF-loaded Phosal 50 PG-based TrEthOs represented lower ranges in the vesicle size of the formulations than that of TDLF-loaded Phosal 53 MCT-based TrEthOs. PG, as a water-miscible organic solvent, aided the emulsification/wetting ability of the phospholipids in TDLF-loaded Phosal 50 PG-based TrEthOs, forming smaller vesicles than TDLF-loaded Phosal 53 MCT-based TrEthOs, composed from the PG-free, more lipophilic Phosal 53 MCT (Hoogevest, 2020; Hsieh et al., 2022). On the other hand, increasing the Phosal 53 MCT amount in nano-dispersions was found to increase the vesicular size (Allam et al., 2015). Another rationale is the possible lipid-solvent interactions, especially between cholesterol and the intrinsic PG of Phosal 50 PG in the lipid phase, leading to smaller vesicular size even with increasing cholesterol or PEG levels in formulations, as evidenced in earlier studies (Ohtake et al., 2006; Wehrle et al., 2024).

#### 3.2.3 Influences on entrapment efficiency percentage (Y_2_)

The outcomes listed in Table 2 revealed that the entrapment efficiency percentage (Y_2_) of the BBD TDLF-loaded Phosal-based TrEthOs formulations was ranged from 47.81 to 94.6% for F7 and F10, respectively. It was observed that the Phosal type utilized in formulating TrEthOs (X_1_) has a significant negative effect on Y_2_, as confirmed in the Pareto chart of Y_2_ (Fig. 1). In F10 and F2, the alteration of X_1_ from Phosal 53 MCT to Phosal 50 PG was accompanied by a decrease in Y2 from 94.6% to 57.66%, respectively, at fixed levels of other factors. It was also noted that both PEG 400 percentage concentrations (X2) and cholesterol percentage concentrations (X3) have a significant synergistic effect on Y_2_. The decrease of X_2_ from 30% to 0% resulted in a decrease in Y2 from 94.6% in F10 to 76.27% in F3, with the other factors held at fixed levels. In addition, it was observed that increasing X3 from 0% to 10% resulted in a rise in Y2 from 75.77% in F8 to 92.84% in F9, while maintaining constant levels of X_1_ and X_2_. The findings demonstrated that the entrapment efficiency of TDLF in the TrEthOs formulations increases with the increase in the amount of PEG 400 used to construct the nanovesicles. This finding was consistent with previous investigations (Al-Zuhairy et al., 2023; Hassanzadeganroudsari et al., 2019; Waddad et al., 2013). Additionally, the utilization of Phosal 53 MCT in TrEthOs formulations results in an increase in entrapment capacity, as previous research has shown that Phosal 53 MCT-based nanovesicles can load high amounts of poorly soluble drugs (Komeil et al., 2021). Additionally, using the more hydrophilic Phosal 50 PG in the formulation (with a 40% PG composition) would potentially decrease vesicular wall rigidity and, consequently, lead to more leaky vesicles and lower entrapment efficiencies (Hoogevest, 2020). Furthermore, the percentage of entrapment efficiency rose with the increase in cholesterol levels. An increase in cholesterol will cause the membrane to become more rigid, leading to a decrease in leaky vesicles and an increase in entrapment efficiency. As a result, cholesterol will form nanovesicular TrEthOs with lower fluidity, resulting in a stiffer texture and fewer leaky vesicles by delaying the transition from the gel to the liquid phase (Abdelbary and El-gendy, 2008; Waddad et al., 2013).

#### 3.2.4 Influences on initial and cumulative TDLF permeation (Y_3_ and Y_4_), and *ex vivo* skin permeation parameters (Y_5_, Y_6_, and Y_7_)

**Fig. 2:**
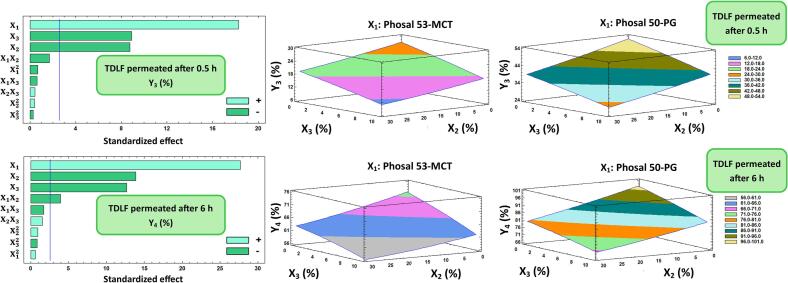
Left panel: Pareto chart displaying the factors' standardized influence on the observed responses (Y_3_ and Y_4_). Mid and right panel: Plots of contour-response surfaces illustrating how variables affect the observed responses (Y_3_ and Y_4_). Note: X_1_ is the Phosal type, X_2_ is the PEG 400 concentration (%), and X_3_ is Cholesterol concentration (%). X_1_X_2_, X_1_X_3_, X_2_X_3_ are the interaction terms between the factors. X_1_^2^, X_2_^2^, X_3_^2^ are the quadratic terms of the factors.

**Fig. 3:**
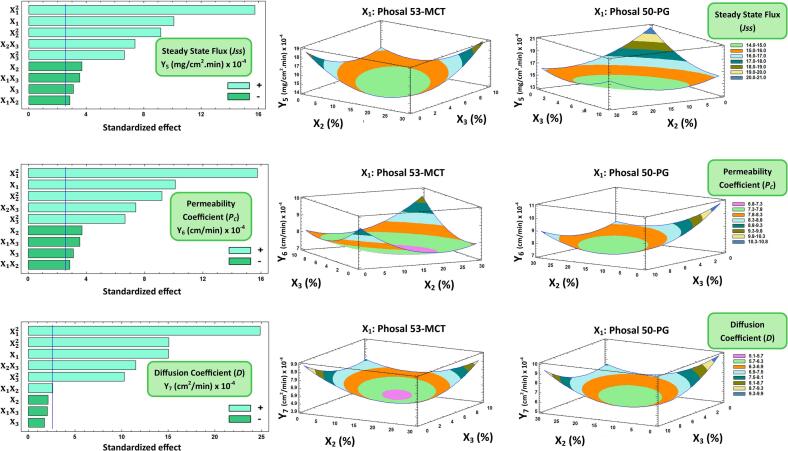
Left panel: Pareto chart displaying the factors' standardized influence on the observed responses (Y_5_, Y_6_, and Y_7_). Mid and right panel: Plots of contour-response surfaces illustrating how variables affect the observed responses (Y_5_, Y_6_, and Y_7_). Note: X_1_ is the Phosal type, X_2_ is the PEG 400 concentration (%), and X_3_ is Cholesterol concentration (%). X_1_X_2_, X_1_X_3_, X_2_X_3_ are the interaction terms between the factors. X_1_^2^, X_2_^2^, X_3_^2^ are the quadratic terms of the factors.

**Fig. 4:**
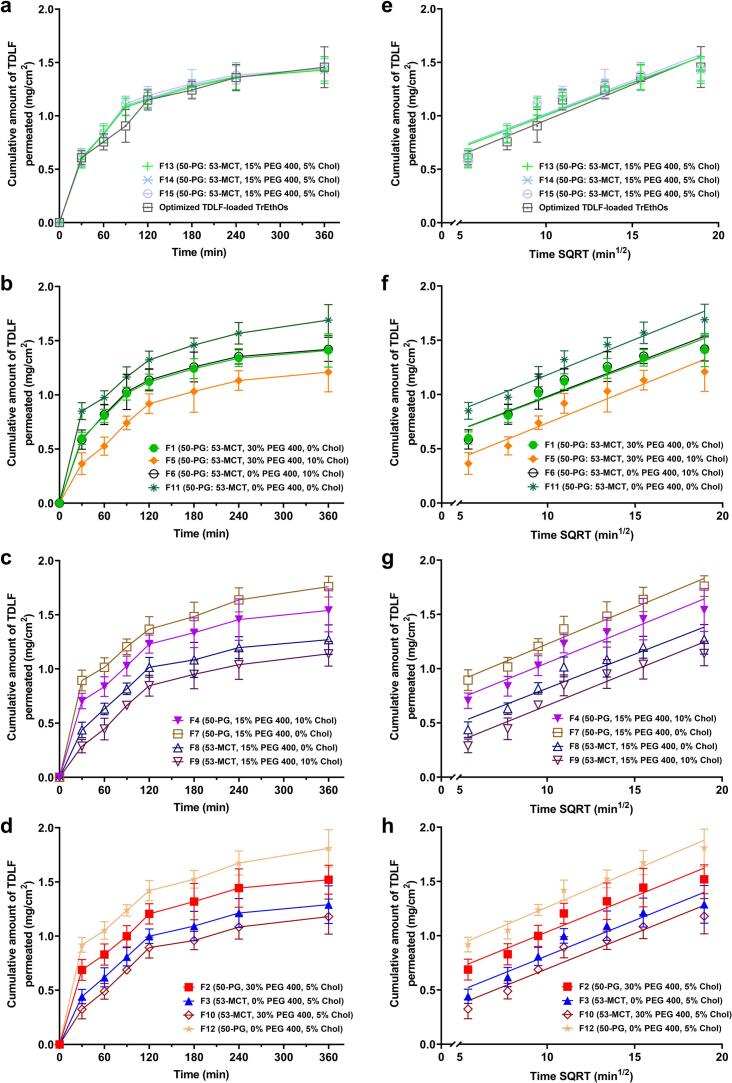
*Ex vivo* permeation pattern of BBD formulations as well as the optimized TDLF-loaded TrEthOs plotted in terms of the cumulative amount of TDLF permeated versus time (left panel), and versus time square root (right panel).

The Pareto charts, along with plots of contour-response surfaces of both initial and cumulative TDLF permeation percentages (Y_3_ and Y_4_, respectively), were displayed in Fig. 2, while those for the *ex-vivo* skin permeation parameters, namely steady-state flux (*J*_*ss*_; Y_5_), permeability coefficient (*Pc*; Y_6_), and diffusion coefficient (*D*; Y_7_), were demonstrated in Fig. 3. Additionally, the initial permeated TDLF amount was observed at 0.5 h, while the cumulative permeated TDLF amount was detected at 6 h, as shown in Fig. 4. Also, the other *ex vivo* skin permeation parameters were extracted from Fig. 4 and displayed in Table 2.

Y_3_ varied from 14.4% for F9 to 45.92% for F12, while Y_4_ ranged from 56.83% for F9 to 90.57% for F12. The Phosal type utilized in the TrEthOs formulation (X_1_) was the main factor synergistically affecting Y_3_ and Y_4_ (Fig. 2). That is, compared to the TDLF-loaded Phosal 53 MCT-based TrEthOs, the TDLF-loaded Phosal 50 PG-based TrEthOs exhibit greater ranges in both the initial and cumulative TDLF permeation percentages of the formulations. In F2 and F10, the alteration in X_1_ from Phosal 50 PG to Phosal 53 MCT was accompanied by a reduction in the percentage of initial TDLF permeated, from 34.4% to 16.22%, respectively. In the same example, a decrease in cumulative permeation percentage from 76.2% to 58.85%, respectively, was also noted with the change in X_1_ from Phosal 50 PG to Phosal 53 MCT at the same levels of X_2_ and X_3_. Using Phosal 50 PG enhanced the permeability of TrEthOs, which was in accordance with an earlier study (Hsieh et al., 2022). Phosal 50 PG was believed to potentially contribute to decreasing the rigidity of the vesicular walls due to the penetration-enhancing ability of its intrinsic PG, resulting in more elastic TrEthOs and higher permeability (Hoogevest, 2020).

Besides, it was observed that both PEG 400 percentage concentrations (X_2_) and cholesterol percentage concentration (X_3_) have significant antagonistic effects on both Y_3_ and Y_4_. The decrease of X_2_ from 30% to 0% yielded an increase in Y_3_ from 34.4% in F2 to 45.92% in F12, at constant levels of X_1_ and X_3_. In addition, it was observed that decreasing X3 from 10% to 0% resulted in an increase in Y4 from 77.23% in F4 to 87.94% in F7, while maintaining fixed levels of the other factors. Moreover, the interaction term (X_1_X_2_) had a negative effect on Y4, Y5, and Y6, while also having a positive impact on Y_7_ (Fig. 3). Additionally, X_2_X_3_ and the quadratic terms of X_1_, X_2_, and X_3_ significantly positively impacted Y_5_, Y_6_, and Y_7_. In contrast, the interaction term (X_1_X_3_) had a negative impact on Y_5_ and Y_6_. These outcomes concluded that the permeation of TDLF-loaded TrEthOs formulations is increased by decreasing both the amount of PEG 400 and cholesterol in TrEthOs nanovesicles. In particular, decreasing cholesterol will lead to more elastic nanovesicular TrEthOs with higher fluidity and, thus, better permeation ability (Abdelbary and El-gendy, 2008; Al-Zuhairy et al., 2023; Hassanzadeganroudsari et al., 2019; Waddad et al., 2013). However, if the cholesterol concentration significantly rose above a specific limit, it would potentially disrupt the bilayer structure of nanovesicles (Kassem et al., 2017), resulting in higher elasticity and improved skin permeability. This was evidenced by the positive impact of the quadratic term of cholesterol percentage concentration (X_3_) on *ex vivo* skin permeation parameters (Y_5_, Y_6_, and Y_7_).

Likewise, the synergistic effect of the quadratic term of PEG 400 percentage concentrations (X_2_) on *ex vivo* skin permeation parameters (Y_5_, Y_6_, and Y_7_) can be mainly attributed to the significant increase in the degree of skin swellability, wettability, and thus diffusivity of TrEthOs. Because low molecular weight PEGs are more hydrophilic than high molecular weight PEGs, they can swell well and permit greater drug diffusion from TrEthOs and through the skin, which further explains the improved wettability of TrEthOs with high levels of PEG 400 (El-Say et al., 2021; Moradkhannejhad et al., 2020).

#### 3.2.5 Mathematical modeling and statistical analysis of the experimental data

Following an examination of the values of all BBD-examined responses, mathematical modeling for each response was produced. Eqs. (3), (4), (5), (6), (7), (8), (9) detail the analytical results of the multiple linear regression analysis using the best-fit method.

(3) $$\text{Mean vesicle size}\;\left(Y_{1}\right)=582.64-230.78\;X_{1}+2.39\;X_{2}+4.4\;X_{3}-82.16\;X_{1}^{2}+2.42\;X_{1}X_{2}+3.16\;X_{1}X_{3}-0.26\;X_{2}^{2}-0.42\;X_{2}X_{3}-1.17\;X_{3}^{2}\;$$

(4) $$\text{Entrapment efficiency}\;\left(Y_{2}\right)=59.28-9.98\;X_{1}-0.27\;X_{2}+0.33\;X_{3}+0.86\;X_{1}^{2}-0.22\;X_{1}X_{2}-0.56\;X_{1}X_{3}+0.02\;X_{2}^{2}+0.05\;X_{2}X_{3}+0.05\;X_{3}^{2}\;$$

(5) $$\text{Percentage of TDLF initial permeation after}\;0.5\;h\;\left(Y_{3}\right)=40.72+12.63\;X_{1}-0.41\;X_{2}-1.02\;X_{3}-0.6\;X_{1}^{2}-0.09\;X_{1}X_{2}-0.1\;X_{1}X_{3}+0.002\;X_{2}^{2}+0.005\;X_{2}X_{3}-0.01\;X_{3}^{2}\;$$

(6) $$\text{Percentage of TDLF cumulative permeation after}\;6\;h\;\left(Y_{4}\right)=83.07+14.22\;X_{1}-0.5\;X_{2}-0.98\;X_{3}+0.35\;X_{1}^{2}-0.15\;X_{1}X_{2}-0.19\;X_{1}X_{3}+0.003\;X_{2}^{2}+0.01\;X_{2}X_{3}-0.02\;X_{3}^{2}\;$$

(7) $$\text{Steady State Flux}\;\left(Y_{5}\right)=18.08+2.71\;X_{1}-0.39\;X_{2}-0.94\;X_{3}+3.28\;X_{1}^{2}-0.04\;X_{1}X_{2}-0.14\;X_{1}X_{3}+0.009\;X_{2}^{2}+0.02\;X_{2}X_{3}+0.06\;X_{3}^{2}\;$$

(8) $$\text{Permeability Coefficient}\;\left(Y_{6}\right)=9.04+1.36\;X_{1}-0.19\;X_{2}-0.47\;X_{3}+1.64\;X_{1}^{2}-0.02\;X_{1}X_{2}-0.07\;X_{1}X_{3}+0.04\;X_{2}^{2}+0.01\;X_{2}X_{3}+0.03\;X_{3}^{2}$$

(9) $$\text{Diffusion Coefficient}\;\left(Y_{7}\right)=8.14+0.99\;X_{1}-0.29\;X_{2}-0.67\;X_{3}+2.55\;X_{1}^{2}+0.02\;X_{1}X_{2}-0.04\;X_{1}X_{3}+0.007\;X_{2}^{2}+0.02\;X_{2}X_{3}+0.04\;X_{3}^{2}\;$$

### 3.3 Prediction and assessment of the optimized TDLF-loaded TrEthOs

An optimized TDLF-loaded TrEthOs can be obtained through multiple response optimization, which meets the necessary objectives of obtaining the lowest possible mean vesicle size and the highest possible values of other permeability attributes assigned for the BBD design, such as the percentage of entrapment efficiency, the percentage of initial TDLF permeated after 0.5 hours, the percentage of cumulative TDLF permeated after 6 hours, steady-state flux, permeability coefficient, and diffusion coefficient. To achieve a combination of characteristics that enhance the desirability function in relation to the study goals, the optimum parameters were identified and examined to balance the responses. The desirability value was found to be 0.68 and the optimized levels of the independent variables were identified as follows: Phosal 50 PG as the optimized Phosal type (X_1_), 30% as the optimized PEG 400 percentage concentration (X_2_), and 10% as the optimized cholesterol percentage concentration (X_3_). The optimized DLF-loaded TrEthOs were then formulated and evaluated, as demonstrated in the previous section (Section 2.4). The detected values of these responses were found to be 129.74 nm, 67.3%, 28.3%, 70.24%, 19.49 × 10^−4^ mg/cm^2^ × min, 9.74 × 10^−4^ cm/min, and 11.16 × 10^−4^ cm^2^/min, for Y_1_, Y_2_, Y_3_, Y_4_, Y_5_, Y_6_, and Y_7_, respectively, while the predicted values were 144.33 nm, 72.8%, 30.4%, 72.8%, 18.03 × 10^−4^ mg/cm^2^ × min, 9.02 × 10^−4^ cm/min, and 9.27 × 10^−4^ cm^2^/min, respectively.

Additional characterization of the optimized TDLF-loaded TrEthOs has been carried out using differential scanning calorimetry (DSC), Fourier transform infrared spectroscopy (FTIR), and morphological analysis. Moreover, the permeation properties of the excised rat skin were further investigated for the optimized TrEthO formulation *via* confocal and cellular uptake *in vitro* studies.

#### 3.3.1 Optical and transmission electron microscope examination

**Fig. 5:**
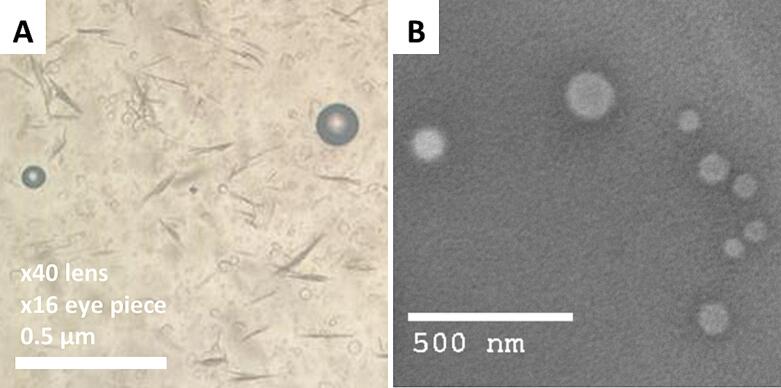
Light (A) and TEM (B) micrographs of the optimized TDLF-loaded TrEthOs.

The generated photomicrographs displayed the spherically formed, bilayer-structured nanovesicles of TrEthOs. The resulting photomicrographs for the optimized TDLF-loaded TrEthOs are shown in Fig. 5. In the micrographs, the vesicular size range matched the previously reported measured range in the section above. The reproducibility and dependability of the TrEthOs synthesis process are highlighted by this size consistency. The effective encapsulation of TDLF within these nanovesicles is further supported by the bilayer structure shown in the micrographs. These morphological features crucially indicate the stability and appropriateness of the formulations for drug delivery applications (El-Say et al., 2024).

#### 3.3.2 FTIR and DSC analysis

**Fig. 6:**
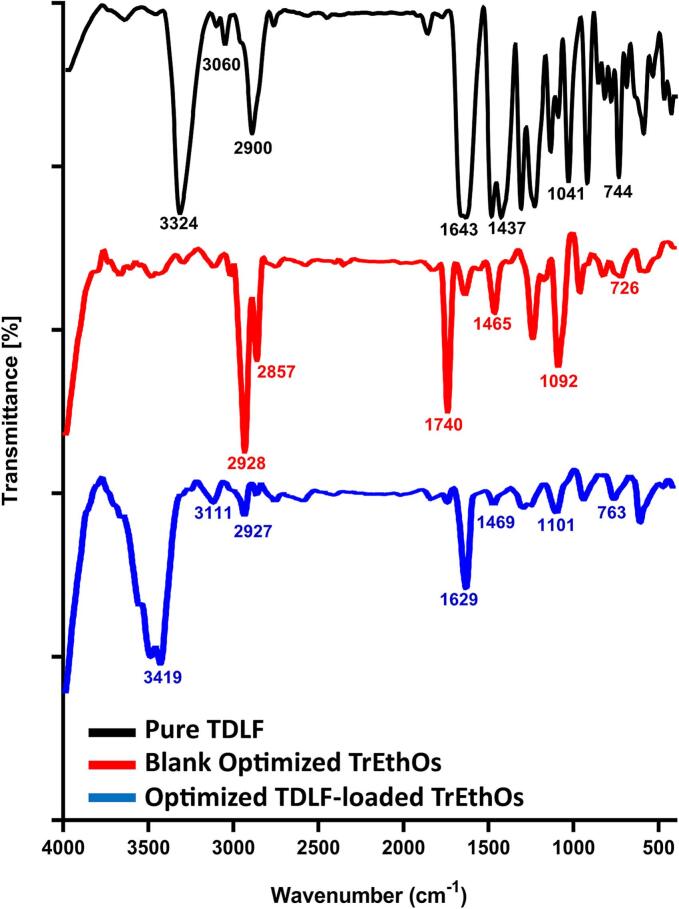
FTIR Spectra of pure TDLF (top panel), blank optimized TrEthOs (middle panel), and the optimized TDLF-loaded TrEthOs (bottom panel).

**Fig. 7:**
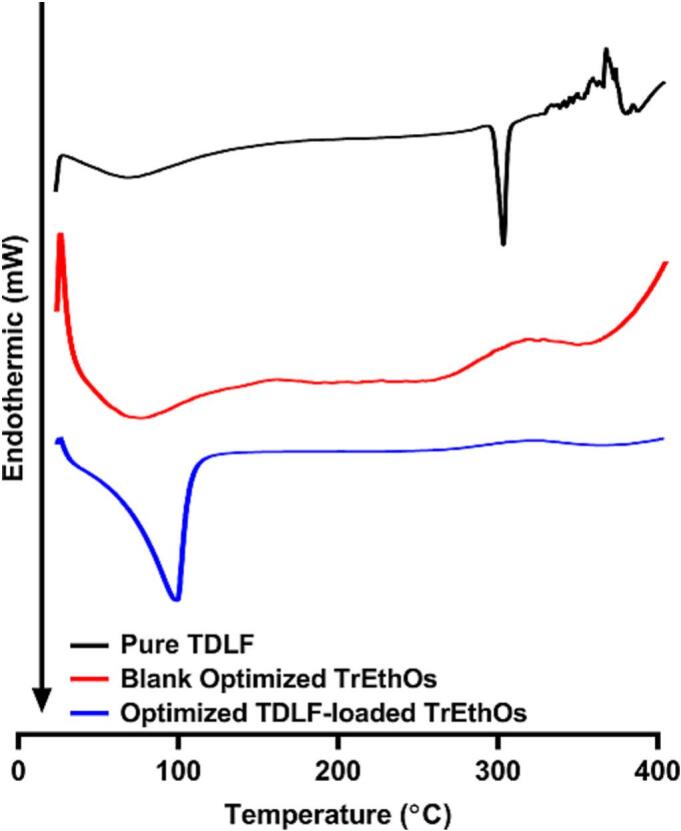
DSC Thermograms of pure TDLF (top panel), blank optimized TrEthOs (middle panel), and the optimized TDLF-loaded TrEthOs (bottom panel).

Fig. 6, Fig. 7 show the FTIR spectra and DSC thermograms, respectively, of the optimized TDLF-loaded TrEthOs, the pure TDLF, and blank optimized TrEthOs.

In accordance with earlier studies, pure TDLF displayed distinct absorption bands that were indicative of the aromatic ring (1473 cm^−1^), amine (3324 and 1643 cm^−1^), and additional bands at 2900, 1437, 1041, 920, and 744 cm^−1^ (Coelho Neto and Lisboa, 2017; Wei et al., 2018; Zou et al., 2006). On the other hand, the blank optimized TrEthOs comprised absorption bands that related to the distinctive peaks of individual components. From literature, representative bands of νO-H (3375.81 cm^−1^) and νC-H Stretching of Methyl and Methylene (2928 cm^−1^ and 2857 cm^−1^) were mostly corresponding to cholesterol (Darson et al., 2023), while bands at 1740 cm^−1^ and 1465 cm^−1^ were consistent with those for Phosal® 50 PG and PEG 400, respectively (Hsieh et al., 2022; Sajjan et al., 2020).

Additionally, it was noted that there was no discernible shifting or interference with the distinctive TDLF peaks in the absorption bands of the optimized TDLF-loaded TrEthOs. The absence of new peaks and the minor shifts in absorption bands in the optimized TDLF-loaded TrEthOs suggest only physical interactions (such as hydrogen bonding) without substantial chemical incompatibility or interaction between TDLF and ingredients used in the optimized formulation.

Regarding DSC, the pure TDLF thermogram exhibited a distinct endothermic peak at 302 °C, corresponding to the TDLF melting point. However, in the DSC thermogram of the optimized TrEthOs loaded with TDLF, the typical peak of TDLF vanished (Fig. 7), displaying a distinctive peak at 101 °C, which is mostly related to PEG 400 (Sajjan et al., 2020) incorporated within the formulation. This was in relation to the corresponding peak represented by the DSC thermogram of the blank formulation, along with other broad peaks at around 150-250 °C corresponding to cholesterol and phospholipids of Phosal® 50 PG (Kalamkar and Wadher, 2019; Kassem et al., 2017). This behavior can be linked to the full interaction between TDLF and the nanovesicular bilayer composition of the optimized TrEthOs formulation. This includes the enhanced encapsulation of TDLF within the TrEthOs formulation (Al-Zuhairy et al., 2023; Gardouh et al., 2021; Hoogevest, 2017; Kalamkar and Wadher, 2019).

All of these results from FTIR and DSC show improved entrapment of TDLF into the optimized TrEthOs formulation and good interactions amongst the constituents of the TrEthOs bilayers.

### 3.4 Confocal laser scanning microscope evaluation

**Fig. 8:**
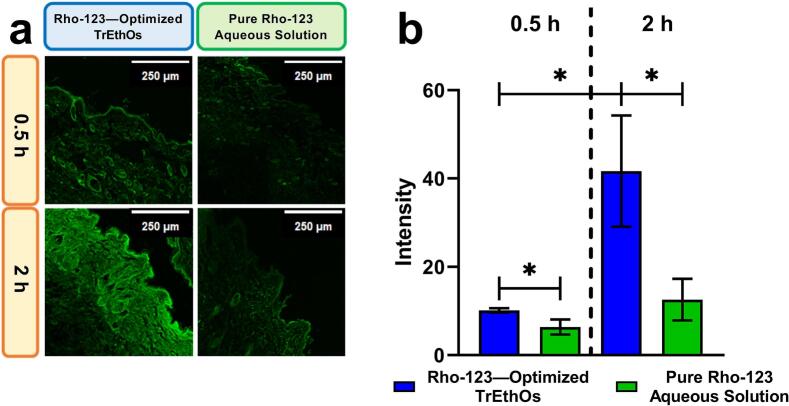
(a) CLSM micrographs showing cross-sections of Wistar rats' skin visualizing the penetration of either Rho-123-loaded optimized TrEthOs or Rho-123 aqueous solution 0.5 and 2 h after application. (b) The amplitude of Rho-123 fluorescence in the chosen CLSM micrographs of the skin cross-sections of Wistar rats that were penetrated by Rho-123 aqueous solution or Rho-123–loaded optimized TrEthOs 0.5 and 2 h after application. At P < 0.05, a significant difference is indicated by an asterisk (*). The optimized TrEthOs loaded with Rho-123 demonstrated a significantly greater fluorescence intensity than the Rho-123 aqueous solution when applied through the layers of the Wistar rats' skin at both 0.5 and 2 h time points.

According to the micrographs of the Wistar rat skin cross-sections captured and analyzed via CLSM (Fig. 8), the optimized TrEthOs loaded with Rho-123 exhibited distinct permeability patterns compared to the pure Rho-123 solution.

At 0.5- and 2-hour intervals after the Rho-123-loaded optimized TrEthOs was applied, the fluorescence intensity (amplitude) was recorded and analyzed, and the outcomes were contrasted with those of the pure Rho-123 solution. The permeability pattern of the pure Rho-123 solution was found to agree with results from previous investigations (El-Say et al., 2024; Troutman and Thakker, 2003). It was also observed that the 0.5 h intensity of the Rho-123–loaded optimized TrEthOs was significantly more than that of the pure Rho-123 solution (P < 0.05). It was also noted that the Rho-123–loaded optimized TrEthOs exhibited a significantly higher 2-h intensity than the 2-h intensity of the pure Rho-123 solution (3.32-folds; P < 0.05) and the 0.5-h intensity of the Rho-123–loaded optimized TrEthOs (4.12-folds; P < 0.05). Remarkably, the optimized TrEthOs loaded with Rho-123 validated the increased skin permeability.

This was thought to be primarily caused by the Phosal 50 PG and PEG 400 incorporated in the optimized TrEthOs formulation, as indicated by the increased intensity of Rho-123 at both periods. TrEthOs' permeability was increased by using Phosal 50 PG, which was consistent with previous research (Hsieh et al., 2022). In addition to the intrinsic PG's capacity in Phosal 50 PG to enhance skin penetration as a permeability enhancer, Phosal 50 PG was also thought to have the potential to help reduce the rigidity of the vesicular walls, which would lead to a more elastic TrEthOs and thus further increased permeability (Hoogevest, 2020).

In summary, the results from these CLSM images showed that the optimized TrEthOs loaded with Rho-123 confirmed the increased skin permeability resulting from the more hydrophilic Phosal 50 PG and PEG 400 in the TrEthOs.

### 3.5 *In vitro* cytotoxicity and cellular uptake investigations of the optimized formulation

#### 3.5.1 Safety of the optimized TDLF-loaded TrEthOs on HSF cell line

**Fig. 9:**
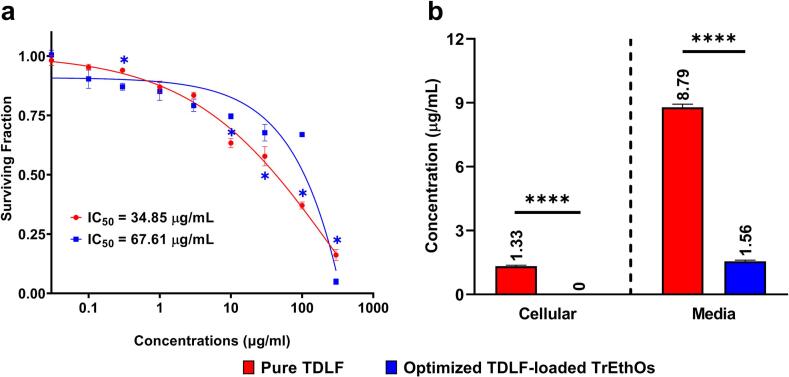
(a) Cytotoxicity of treatment of pure TDLF and the optimized TDLF-loaded TrEthOs in HSF cell line after 48 h (surviving fraction and IC_50_ concentrations). The results are expressed as the mean ± SD (n = 3). Note: * denotes a significant difference between pure TDLF and the optimized TDLF-loaded TrEthOs at P < 0.05. (b) Cellular uptake of pure TDLF and the optimized TDLF-loaded TrEthOs after 4 h interval in HSF cell line. The results are expressed as the mean ± SD (n = 3). Note: **** denotes a significant difference between pure TDLF and the optimized TDLF-loaded TrEthOs at P < 0.0001.

Following a 48-hour incubation period with both pure TDLF and the optimized TDLF-loaded TrEthOs, the MTT assay was used to assess the viability and proliferation of HSF cells (Fig. 9).

IC_50_ values for a 48-h exposure period were calculated, and the mean inhibitory effects of pure TDLF and the optimized TDLF-loaded TrEthOs on the proliferation of HSF cells were identified. The findings demonstrated that unloaded plain nanovesicles had no discernible effect on the number of colonies or the percentage of viability compared to control cells, which had a viability of 99.64%. As a result, at all equal concentrations, the plain nanovesicles had no discernible inhibitory effect on HSF cells.

The findings also showed that, especially at high doses, the survival fraction trend of HSF cells treated with pure TDLF was considerably lower than that of the optimized TDLF-loaded TrEthOs (P < 0.0001, Fig. 9).

The values of IC_50_ in HSF cells treated with pure TDLF and the optimized TDLF-loaded TrEthOs were 34.85 and 67.61, respectively, which reflect the superior safety of the optimized TDLF-loaded TrEthOs over the pure TDLF on normal skin cells (Wiranowska et al., 2020). However, the cell viability study conducted here did not exclude or reflect the probability of high internalization of the optimized TDLF-loaded TrEthOs. To confirm the preferential internalization and reflect intracellular kinetics and activity of the optimized TDLF-loaded TrEthOs versus pure TDLF, further investigation (*via* cellular uptake study) was performed and interpreted.

#### 3.5.2 Cellular uptake and internalization of the optimized formulation in HSF cell line compared to pure TDLF

The optimized TDLF-loaded TrEthOs' internalization feature was verified through cellular uptake using an HSF cell line. To compare the cellular uptake of pure TDLF and TDLF-loaded TrEthOs under identical conditions, HSF cells were incubated for 4 h with a 50 μg/mL concentration of each. This concentration was selected based on the need to standardize dosing and ensure detectable drug uptake. Although it exceeds the 48 h IC₅₀ of pure TDLF, the short exposure duration (4 h) minimizes cytotoxicity during the assay, allowing accurate assessment of uptake before significant cell death occurs. The cellular concentration of pure TDLF after a 4-h interval was 1.33 ± 0.043 μg/mL, as shown in Fig. 9. In contrast, the cellular concentration of the optimized TDLF-loaded TrEthOs was insufficient to be identified (significant difference from pure TDLF; P < 0.0001). Interestingly, on the other hand, the concentration of pure TDLF in the media was 5.64 times greater than that of the optimized TrEthOs loaded with TDLF (P < 0.0001). These outcomes emphasized that much more TDLF-loaded TrEthOs were internalized within the cells than the pure TDLF, and the decrease in the cellular concentration of the optimized TDLF-loaded TrEthOs formulation after cellular uptake is likely due to efficient internalization, intracellular release, degradation of the nanocarriers, lysosomal activity, metabolic processing, and potential rapid clearance or exocytosis. These processes collectively contribute to the reduced detectable concentration of the formulation within the cells. The primary cause of this type of cell-nanocarrier interaction may be correlated with internalization through rapid and nonspecific phagocytosis (Kassem et al., 2017; Shaker et al., 2015; Xie et al., 2020). Although TDLF has a known systemic half-life of approximately 17.5 h, it was not detected in HSF cells after 4 h of exposure. This discrepancy is likely due to differences between *in vivo* and *in vitro* environments. In systemic circulation, TDLF is extensively protein-bound and cleared primarily by hepatic metabolism, contributing to its long half-life (Coward and Carson, 2008).

In contrast, within HSF cells, the drug is unbound and directly exposed to intracellular enzymes, which may metabolize the compound more rapidly. This intracellular biotransformation, combined with the absence of protective systemic factors, can lead to faster degradation. Additionally, limitations related to the sensitivity of the HPLC method, possible losses during cell lysis, and incomplete extraction may have contributed to the non-detection of TDLF, especially at lower intracellular concentrations or after partial breakdown. Despite this, the observed differences in cellular uptake between the pure drug and the formulation remain relevant and reflect improved internalization by the TrEthO delivery system.

To conclude, the cellular uptake study confirmed that HSF cells internalized the optimized TDLF-loaded TrEthOs more efficiently than pure TDLF, suggesting that the nanocarrier system facilitates better intracellular delivery and potential activity. These findings highlight the potential of TDLF-loaded TrEthOs as a promising approach for transdermal drug delivery, offering improved safety and efficacy. To better understand the intracellular behavior of the optimized TDLF-loaded TrEthOs, future studies should focus on the intracellular kinetics, including the release profile of TDLF from the nanocarriers and its interaction with cellular components such as lysosomes or enzymes. Additionally, although the current study focused on formulation development and ex vivo evaluation, in vitro release profiles, short-term, and long-term stability assessments were not conducted. While *in vitro* drug release studies are commonly used to assess release kinetics in early-stage formulation development, they do not account for skin barrier interactions and were not considered within the defined scope of this work. Instead, we prioritized *ex vivo* evaluation, which provides a more biologically relevant assessment of the drug delivery potential through the skin. Similarly, stability studies were not performed, as the aim was to focus on evaluating formulation behavior and performance under experimental conditions rather than long-term storage outcomes. However, evaluating both the *in vitro* release profile and the storage stability of the formulation is essential in our future studies to validate its potential for practical application, ensure reproducibility, and support further preclinical development.

## 4 Conclusion

This study presents a Phosal-based transethosomal (TrEthOs) system as an advanced strategy for enhancing the transdermal delivery of tadalafil (TDLF). The optimized formulation, developed using a Box-Behnken Design, demonstrated favourable characteristics for transdermal drug delivery. The system showed successful entrapment, morphological stability, and optimized *ex vivo* skin permeation, supporting its potential to improve the transdermal delivery of poorly soluble therapeutic agents. FTIR and DSC analyses confirmed physical compatibility between the drug and excipients. The study also demonstrated that the optimized TDLF-loaded TrEthOs formulation exhibited superior safety and cellular uptake compared to pure TDLF in Human Skin Fibroblast (HSF) cells. Confocal microscopy further validated the deep skin penetration of the optimized TrEthOs. The overall findings emphasized the superiority of the optimized TDLF-loaded TrEthOs over pure TDLF in terms of safety, activity, and transdermal/cellular permeability, suggesting an auspicious approach for the efficient transdermal delivery of TDLF and suitability for further development. Despite these promising findings, our future considerations will include additional research on intracellular kinetics, *in vivo* pharmacodynamics, and pharmacokinetics. Although *in vitro* release and stability studies were not included in our recent work (which might be subconsciously considered as a limitation of this work), the *ex vivo* permeation model provided direct insight into the system’s ability to enhance skin delivery, which was the primary objective of the current work. Future studies may incorporate these aspects to provide a broader evaluation of the formulation's performance under various conditions. In addition, preclinical and clinical efficacy in animal and human trials, long-term stability studies, and feasibility of large-scale production are all necessary to assess real-world efficacy and patient compliance and to translate this technology into pharmaceutical drugs with potential for commercialization. Future work should also explore targeted TrEthOs formulations for other poorly soluble drugs, expanding the potential of this innovative transdermal drug delivery approach. This research advances transdermal therapeutics, offering a controlled-release system with enhanced bioavailability and improved patient adherence.

## CRediT authorship contribution statement

**Turky Omar Asar:** Validation, Resources. **Hossam S. El-Sawy:** Writing – review & editing, Writing – original draft, Visualization, Validation, Methodology, Investigation, Formal analysis. **Ahmed M. Reda:** Writing – original draft, Validation, Formal analysis. **Mohamed Ashraf:** Validation, Methodology, Investigation, Formal analysis. **Amer H. Asseri:** Validation, Resources. **Abdelsattar M. Omar:** Validation, Resources. **Tarek A. Ahmed:** Writing – review & editing, Validation. **Khalid M. El-Say:** Writing – review & editing, Validation, Supervision, Resources, Project administration, Formal analysis, Conceptualization.

## Ethics approval

Rat skin was prepared for *ex vivo* research in accordance with previously published literature following ethical approval by the Egyptian Russian University Faculty of Pharmacy's Ethical Committee (ERUFP-PT-24-001). The handling of animals and the ex vivo studies have been conducted in compliance with Animal Research: Reporting of In Vivo Experiments (ARRIVE) guidelines. In addition, the study has also been performed in accordance with the National Institutes of Health guide for the care and use of Laboratory animals (NIH Publications No. 8023, revised 1978). The animals had unrestricted access to water. General conditions and the environment were closely monitored.

## Declaration of generative AI and AI-assisted technologies in the writing process

During the preparation of this work, the authors used QuillBot/Paraphraser (Standard/free version) as little as possible to improve readability and language solely. After using this tool/service, the authors reviewed and edited the content as needed and took full responsibility for the publication's content.

## Funding

This work was funded by the University of Jeddah, Jeddah, Saudi Arabia under grant No. (UJ-23-SRP-6).

## Declaration of competing interest

The authors declare that they have no competing interests.

## Data Availability

Data will be made available on request.
